# The Fifteenth International Congress of Medicine

**Published:** 1906-04-28

**Authors:** 


### HOSPITAL ADMINISTRATION.

### CONSTRUCTION AND ECONOMICS.

### THE FIFTEENTH INTERNATIONAL CONGRESS OF MEDICINE.

From our own Correspondent.

The instincts of race are obliteratel with difficulty even by years of civilisation. English, Scots, and Irish are naturally vagabonds; of course, in the strictly etymological sense of the word. Their instinct of wandering is plainly seen in the eagerness with which they flock to congresses held in distant places which promise a change of scene with the discomforts of a picnic. Like all northern races, too, they love to migrate with the impedimenta of wives and children, and thus to invade the territories of those whom they look upon as living a life of inglorious ease in the sunny south.

The English contingent who are visiting the International Congress of Medicine, which is now being held at Lisbon, have shown all these curious traits of character. Some have gone by train—a long, dusty, and wearisome route even by the best of trains, the Sud express of the International Sleeping Car Company; others have travelled by the Booth Line from Liverpool, and have found themselves accommodation of a more less agreeable character in Lisbon; the larger number left the Tilbury Docks in the ss. *Ophir*, of the Orient Pacific Line, on the day before Good Friday. No less than 113 have selected this method of travel, and have thus freed themselves from the trouble of hôtels, though they subjected themselves to the annoyance of sea-sickness. The party promised itself some relaxation before beginning the serious work of the Congress, and started with a will to enjoy everything from the beginning.

A sunny day in the Channel and just sufficient ground swell in the Bay of Biscay to remind those inclined to sea-sickness of the depth of despair to which they might have been reduced under less favourable conditions brought the party to Vigo on Easter Sunday. Here a landing was speedily effected, and the lovely land-locked harbour was seen to the best advantage, whilst many gained their first experience of a Spanish town with its bright colours, sorry mules, odd smells, and handsome inhabitants.

By four o'clock the ss. *Ophir* had weighed her anchor and was steaming for Tangier through a calm sea and under a cloudless sky. Monday gave the horde time to organise itself. Money was collected for sports, dances were arranged, bridge and progressive whist parties were formed, and many there were who missed the sight of Cape St. Vincent, owing to the seductions of cards in the drawing-room. By this time the party had become a large family with common wishes and a remarkable unanimity of will. A deputation waited on the Commander to ascertain whether it would be possible to visit Tangier as well as Gibraltar, and there was loudly expressed satisfaction when the reply proved favourable, for it showed that Gibraltar had a clean bill of health—at least, officially.

An early start enabled Tangier to be seen under the unusually good condition of a thunder shower, which laid the dust and mitigated the smells of a most unsanitary town. Many visited the town and outskirts on mules, and marvelled at the cactus hedges, the giant geraniums, and the profusion of wild flowers, whilst others contented themselves with rubbing shoulders with a thoroughly noisome crowd. The passengers were all re-embarked by twelve o'clock, and the *Ophir* proceeded on her way to Gibraltar, which was reached as soon as lunch was finished.

A pleasant afternoon at Gibraltar was followed by twenty hours' open sea until the *Ophir* anchored in the Tagus on Wednesday evening, April 18. The Congress arrangements on the following day were found to be admirable. The Executive Committee, under the guidance of Professor Miguel Bombarda, the active and genial General Secretary, had successfully avoided the crush for tickets which was so discreditable a feature of the Madrid Congress. The meetings and the various offices were installed in the new medical school, a magnificent building which has just been built in the higher part of Lisbon about a mile and a half from the quays. Here each nation or group of nations was allotted a corner in an open courtyard. In each corner was a table presided over by a young lady who was a native of the country with which she had to deal and who was an inhabitant of Lisbon. The tickets and insignia were thus distributed rapidly and without any of the friction due to mutual ignorance of language and customs. The opening ceremony was held in the large hall of the Geographical Society in the presence of their Majesties King Carlos and Queen Maria, and under the presidency of Counsellor Costa Alemanao. It was, as usual, a somewhat dull function of twenty or more speeches delivered for the most part in French, though Sir Dyce Duckworth, the British representative, spoke in English. The evening was devoted to a reunion of the members of the Congress in the Medical School, when the members renewed past friendships and made new acquaintances.

Friday morning was spent in sectional work, the various official reports being read and discussed, but numbers of the members of Congress left at eleven o'clock for the special trains to Cintra, where, by the hospitality of Sir Frederick Cook, they were entertained at Montserrati, one of the most charmingly situated seats in Portugal. Saturday, in the same manner, was occupied with sectional meetings, several sections combining to take part in a general discussion on some points of material interest—notably the section of radium, surgery and urinary surgery which dealt exhaustively with the diagnosis of disease of the kidneys.

